# Dynamic coordination mechanism in single-atom sensing

**DOI:** 10.1093/nsr/nwaf146

**Published:** 2025-04-26

**Authors:** Tao Zhang

**Affiliations:** Dalian Institute of Chemical Physics, Chinese Academy of Sciences, China

Gas sensing technology is crucial for a wide variety of applications [1]. Currently, traditional gas sensing materials are hindered by limitations in selectively detecting single-type gas due to the non-specific interactions between gas molecules and sensing materials [2,3]. Single atoms (SAs) have the potential to generate specific effects on a single-type gas due to the uniform active center configuration [4,5]. However, the means to stimulate the capacity of single atoms towards a single-type gas sensing are still lacking. Therefore, the design of highly sensitive and selective sensing single-atom materials for single-type gas detection needs more attention.

**Figure 1.**
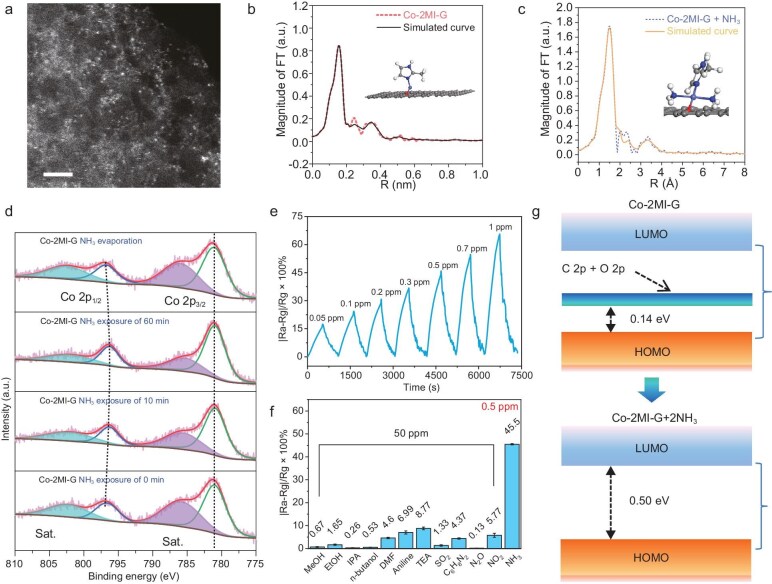
(a) HAADF-STEM image of Co-2MI-G. (b, c) FT-EXAFS curves of the proposed structure and the measured Co-2MI-G and Co-2MI-G + NH_3_. Insets are the proposed model of Co-2MI-G and Co-2MI-G + NH_3_ architectures. (d) Quasi *in-situ* XPS spectra of Co-2MI-G before and after exposure to NH_3_, followed by NH_3_ evaporation. (e) The resistance response curves of the Co-2MI-G-based sensor. (f) Selectivity of Co-2MI-G to NH_3_ and other VOCs gases. (g) Demonstration of frontier molecular orbital structures of Co-2MI-G and Co-2MI-G + 2NH_3_. Reproduced from ref. [6] with permission.

Recently, by utilizing the dynamic changes in the coordination structure of cobalt single atoms, Wu and Feng *et al.* realized high sensitivity and high selectivity towards ammonia gas sensing. In this work, they developed a cobalt single-atom material with a bidentate coordination structure (Co-2MI-G), which exhibited ultra-high selectivity for NH_3_ sensing [6]. A high-angle annular darkfield scanning transmission electron microscopy (HAADF-STEM) image showed that no aggregates were formed on the surface of graphene-like Co-2MI-G and the Co single atoms were uniformly dispersed (Fig. 1a). It is indicated that from the fitting of Fourier transformed-extended X-ray absorption fine structure (FT-EXAFS), the coordination number of Co single atoms changed from 2 in pristine Co-2MI-G to 4 after NH_3_ gas exposure, corresponding to a stable model of bonding with two NH_3_ molecules (Fig. 1b and c). In order to further clarify the special coordination evolution mechanism, quasi *in-situ* X-ray photoelectron spectroscopy (XPS) technology was used to explore the state changes of Co single atoms before and after exposure to NH_3_. As illustrated in Fig. 1d, the satellite peak intensity for Co 2p decreased significantly, while the 2p_1/2_–2p_3/2_ separation energy weakened, indicating the conversion of Co^2+^ to Co^3+^. As a result, the Co-2MI-G-based sensor showed ultra-high sensitivity (67.598% for 1 ppm NH_3_, Fig. 1e) and high selectivity for volatile organic compounds (VOCs) and other interfering gases (Fig. 1f). Theoretical simulation elucidated the chemisorption process of NH_3_ molecules on Co-2MI-G. Density of state (DOS) analysis indicated that the Co-2MI-G adsorbed with NH_3_ displayed a stronger insulation and higher oxidation state than pristine Co-2MI-G, which enlarged the band gap from 0.14 eV to 0.50 eV (Fig. 1g).

In summary, this paper verifies that single atoms with a dynamically changing coordination structure could be used as a single-type gas sensing material with superior sensitivity and selectivity. This work pioneers a novel application of single atoms to the field of single-type gas sensing and provides an innovative perspective on utilizing single atoms by dynamic coordination structure.
